# Chl1 DNA helicase and Scc2 function in chromosome condensation through cohesin deposition

**DOI:** 10.1371/journal.pone.0188739

**Published:** 2017-11-29

**Authors:** Donglai Shen, Robert V. Skibbens

**Affiliations:** Department of Biological Sciences, Lehigh University, Bethlehem, Pennsylvania, United States of America; Florida State University, UNITED STATES

## Abstract

Chl1 DNA helicase promotes sister chromatid cohesion and associates with both the cohesion establishment acetyltransferase Eco1/Ctf7 and the DNA polymerase processivity factor PCNA that supports Eco1/Ctf7 function. Mutation in *CHL1* results in precocious sister chromatid separation and cell aneuploidy, defects that arise through reduced levels of chromatin-bound cohesins which normally tether together sister chromatids (*trans* tethering). Mutation of Chl1 family members (BACH1/BRIP/FANCJ and DDX11/ChlR1) also exhibit genotoxic sensitivities, consistent with a role for Chl1 in *trans* tethering which is required for efficient DNA repair. Chl1 promotes the recruitment of Scc2 to DNA which is required for cohesin deposition onto DNA. There is limited evidence, however, that Scc2 also directs the deposition onto DNA of condensins which promote tethering in *cis* (intramolecular DNA links). Here, we test the ability of Chl1 to promote *cis* tethering and the role of both Chl1 and Scc2 to promote condensin recruitment to DNA. The results reveal that *chl1* mutant cells exhibit significant condensation defects both within the rDNA locus and genome-wide. Importantly, *chl1* mutant cell condensation defects do not result from reduced chromatin binding of condensin, but instead through reduced chromatin binding of cohesin. We tested *scc2-4* mutant cells and similarly found no evidence of reduced condensin recruitment to chromatin. Consistent with a role for Scc2 specifically in cohesin deposition, *scc2-4* mutant cell condensation defects are irreversible. We thus term Chl1 a novel regulator of both chromatin condensation and sister chromatid cohesion through cohesin-based mechanisms. These results reveal an exciting interface between DNA structure and the highly conserved cohesin complex.

## Introduction

Structural changes to the genome that occur over the cell cycle are fundamental yet mysterious features that underlie many cellular events. During G_1_ phase of the cell cycle, chromatin compaction and higher order DNA assemblies termed TADS (topological associated domains) are largely regional [1], [2]. These *cis*-based (intramolecular) and *trans*-based (intermolecular) tetherings of DNA segments must remain dynamic to allow for plasticity and appropriate transcriptional responses to external cues such as changes in temperature, nutrient levels and signaling factors [1], [3–5]. During S phase, *trans* tethers are established specifically between the products of chromosome replication, termed sister chromatids. These *trans* tethers remain stable and thus identify chromatids as sisters until anaphase onset. *Cis* tethers established during prophase also are stable—maintaining fully condensed and disentangled chromosomes through mitosis. These *cis* tethers are required for high fidelity chromosome segregation and the positioning of chromosomes away from the cytokinetic furrow. In an impressive coopting of function through evolution, each of these tethering activities in combination are mediated by SMC (stability of minichromosomes or structural maintenance of chromosomes) complexes that include cohesins (Smc1, Smc3, Mcd1/Scc1/RAD21, Pds5, Scc3/Irr1/SA1,2 and Sororin in vertebrate cells) and condensins (Smc2/Cut14, Smc4/Cut3, Ycs4/Cnd1/DPY-28, Ycg1/Cdn3/CAP-G1, Brn1/Cdn2/DPY-26) [1], [2], [6], [7].

Divisions between SMC complex functions are not always distinct. For instance, it is well established that cohesins form both *cis* and *trans* tethers that function in DNA replication, repair, chromosome segregation, chromatin condensation and transcription regulation [1], [2]. Thus, mutations of cohesin pathways produce aneuploidy, are tightly correlated with numerous cancers and directly result in severe developmental maladies that include Robert Syndrome, Cornelia de Lange Syndrome and Warsaw Breakage Syndrome [2], [8], [9]. Condensins on the other hand, which primarily tether DNA segments in *cis* conformations, provide for longitudinal chromatin compaction, removal of DNA catenations, chromosomal disentanglement, and dosage compensation [6], [7]. Mutations of condensation pathways result in T cell lymphomas, colon cancer, microcephaly, and are predictors of cancer survivorship [10–14]. Mechanistically, convincing evidence suggests that both cohesins and condensins entrap individual DNA segments within a topologically closed structure. In turn, DNA segment tethering requires oligomerization of the appropriate SMC complexes, although little is known regarding how these oligomerization steps are directed toward either *cis* or *trans* conformations [1], [15–17].

The targeting and deposition of cohesins and condensins onto DNA represents a critical regulatory mechanism that spans a wide range of cellular activities, but remains largely undefined. What is clear is that cohesin deposition onto DNA requires the loader complex comprising Scc2/NIPBL and Scc4/MAU-2 [18–22]. One particular study, however, implicated Scc2,4 in the recruitment of condensin to DNA, a finding largely based on fluorescent intensity levels performed on chromosome spreads [23]. In yeast, Scc2,4 recruitment to DNA is regulated at the level of DNA structure and requires the conserved Chl1 DNA helicase [24–26]. At least during S phase, Scc2 deposition appears coordinated with DNA replication fork progression given that Chl1 physically interacts with numerous DNA replication fork factors (PCNA, Rad27/FEN1, MCMs) and the S phase acetyltransferase Eco1/Ctf7 [24], [25], [27–30]. Thus, Chl1 DNA helicase appears as the earliest regulator identified to date of Scc2 and cohesin recruitment to DNA.

Despite the wealth of evidence that Chl1 is critical for sister chromatid *trans*-tethering [27], [31–34], a role for Chl1 in *cis*-tethering remains untested. The issue is a critical one given that mutations in the Chl1 human homologs ChlR1/DDX11 and BACH1/BRIP1/FANCJ collectively result in Warsaw Breakage Syndrome, Fanconi anemia, cell aneuploidy and breast and ovarian cancers [27], [31], [32], [34–40]. Moreover, the extent to which Chl1 DNA helicase regulation of Scc2 translates to both cohesin and condensin recruitment to chromatin is unknown, revealing a significant deficit in our understanding of these clinically relevant processes. Here, we report that Chl1 and Scc2 are indeed regulators of genome-wide condensation, but that these roles occur independent of condensin binding to DNA and instead rely primarily on cohesin function.

## Materials and methods

### Yeast strains and strain construction

*Saccharomyces cerevisiae* strains used in this study are listed in Table 1. GFP-tagging and deletion of genes were performed following a published protocol [41].

**Table 1:** Yeast strains used in this study.

Strain name	Genotype	Reference
**YBS1019**	*MATa; S288C*	[25]
**YBS1041**	*MATa; chl1*::*KAN; S288C*	[27]
**YBS2020**	*MATa; NET1*:*GFP*:*HIS3; w303*	For this study
**YBS2078**	*MATa; lacOs*::*YLR003c-1; lacOs*::*MMP1; LacI-GFP; w303*	Y2869 [23]
**YBS2079**	*MATa; chl1*::*TRP; lacOs*::*YLR003c-1; lacOs*::*MMP1; LacI-GFP; w303*	For this study
**YBS2080**	*MATa; NET1*:*GFP*:*HIS3; chl1*::*TRP; w303*	For this study
**YDS101**	*MATa; SMC2*:*3HA*:*KanMX6; w303*	For this study
**YDS104**	*MATa; SMC2*:*3HA*:*KanMX6; chl1*::*TRP; w303*	For this study
**YDS108**	*MATa; SMC2*:*3HA*:*KanMX6; scc2-4; w303*	For this study
**YMM551**	*MATa; scc2-4; can1-100; w303*	[91]

### rDNA condensation assays

rDNA condensation assays were performed using Net1-GFP as previously described with the following modifications [23], [42]. Briefly, cells were cultured to log phase (OD600 between 0.2 to 0.4), then incubated for 2.5 hours at 23°C in rich YPD medium supplemented with alpha-factor. The resulting synchronized G1 cells were collected, washed, resuspended in fresh YPD supplemented with nocodazole, and incubated for 3 hours at 23°C. The resulting preanaphase cells were fixed by incubation in 3.7% paraformaldehyde for 10 min at 30°C. GFP signals were then assayed microscopically. Cell cycle progression was confirmed by detection of DNA content using flow cytometry as described [42].

rDNA condensation was independently assessed using a streamlined condensation assay adapted from a published FISH protocol [43–45]. Briefly, log phase cells (OD600 between 0.2 to 0.4) were incubated for 2.5 hours at 23°C in rich YPD medium supplemented with alpha-factor. The resulting synchronized G1 cells were collected, washed, resuspended in fresh YPD supplemented with nocodazole, and incubated for 3 hours at 23°C (where appropriate, additional temperature shifts are described within each experimental design). The resulting preanaphase cells were fixed by incubation in 37% formaldehyde for 2 hours at 23°C. Cells were washed with distilled water and resuspended in buffer (1 M sorbitol, 20 mM KPO4, pH 7.4), then spheroplasted by the addition of beta-mercaptoethanol and Zymolyase 100T and incubation for 1 hour at 23°C. The resulting spheroplasted cells were placed onto poly-L-lysine coated slides prior to addition of 0.5% Triton X-100 and 0.5% SDS solution. The slides were then incubated in 3:1 methanol:acetic acid solution and stored at 4°C until completely dry. Slides were then treated with RNase in 2X SSC buffer (0.3 M NaCl, 30 mM Sodium Citrate, pH 7.0) followed by washes in 2X SSC and then by a series of cold ethanol washes. Slides were then incubated at 72°C in 70% formamide with 2X SSC followed by a series of cold ethanol washes. DNA masses were detected by DAPI staining and assayed microscopically. Cell cycle progression was confirmed by detection of DNA content using flow cytometry as described [42].

### Chromosome arm condensation assay

Chromosome arm condensation assays were performed in yeast cells that contained two lacO repeats, one integrated telomere-proximal on the left arm and another integrated centromere-proximal on the right arm of chromosome XII. Each LacO cassette was monitored microscopically through detection of lacI-GFP [23]. Condensation assays and quantification were performed as previously described with the following modifications [23]. Briefly, log phase cells (OD600 between 0.2 to 0.4) were incubated for 2.5 hours at 23°C in rich YPD medium supplemented with alpha-factor. The resulting synchronized G1 cells were collected, washed, resuspended in fresh YPD supplemented with nocodazole and incubated for 3 hours at 23°C. The resulting preanaphase cells were fixed by incubation in 3.7% paraformaldehyde for 10 min at 30°C. Wildtype and *chl1* mutant cells that contained a single GFP dot reflected sister chromatids positioned vertical to the z-axial focal plane and were thus excluded from analysis. We also excluded cells that contained three or four GFP loci, since the cohesion defect at one or both loci made it difficult to determine which GFP dot represented an intra- or inter-sister chromatid locus. Distances between two GFP dots were quantified microscopically with images captured using iVision. Cell cycle progression was confirmed by detection of DNA content using flow cytometry as described [42].

### Chromatin binding assay

Nocodazole arrested cells were harvested and processed for chromatin binding assay [25]. Briefly, the densities of 50 ml cultures were normalized to an OD600 between 0.4–0.6. Cells were spun down and washed with 25 ml cold sterile water, followed by a wash in 1.2 M sorbitol. Pellets were resuspended in 1 ml CB1 buffer (50 mM Sodium citrate, 40 mM EDTA, 1.2 M sorbitol, pH 7.4) prior to the addition of 125 μl of spheroplast solution (125 μl CB1, 50 μl zymolase, 5 μl BME) and incubation with gentle shaking for 1 hour at 23°C. The spheroplast suspensions were supplemented with protease inhibitor cocktail, washed 2X with 1.2 M cold sorbitol, resuspended in 425 μl of 1.2 M cold sorbitol and snap frozen in liquid Nitrogen. Frozen samples were thawed on ice prior to the addition of 50 μl lysis buffer (500 mM Lithium acetate, 20 mM MgSO4, 200 mM HEPES, pH 7.9) and 20 μl of 25% Triton-X-100. Whole cell extract (WCE) fractions were collected and denatured by the addition of an equal volume of 2X Laemmli buffer, boiled for 5 minutes and then snap frozen. The remaining lysates were centrifuged at 12,000 g for 15 minutes. Supernatants consisting of soluble fractions were collected and denatured by the addition of an equal volume of 2X Laemmli buffer. Pellets were resuspended in Lysis buffer with 150 mM NaCl and centrifuged at 15,000 g for 15 minutes. Chromatin bound fractions were obtained by suspending the resulting pellets 1.2 M sorbitol and then denatured by the addition of an equal volume of 2X Laemmli buffer. Whole cell extract, soluble and chromatin bound fractions were resolved by SDS-PAGE electrophoresis and analyzed by Western blot using anti-HA (1:2000) (Santa Cruz), anti-PGK (1:20000) (Invitrogen) with goat anti mouse HRP (1:50000) (Bio-Rad) or by anti-H2B (1:2000) (Santa Cruz) or 1:60000 (Abcam), anti-Mcd1 [46] in combination with goat anti rabbit HRP (1:50000) (Bio-Rad) and ECL prime (GE Healthcare) for visualization.

## Results

### Chl1 DNA helicase promotes rDNA condensation

**Fig 1:**
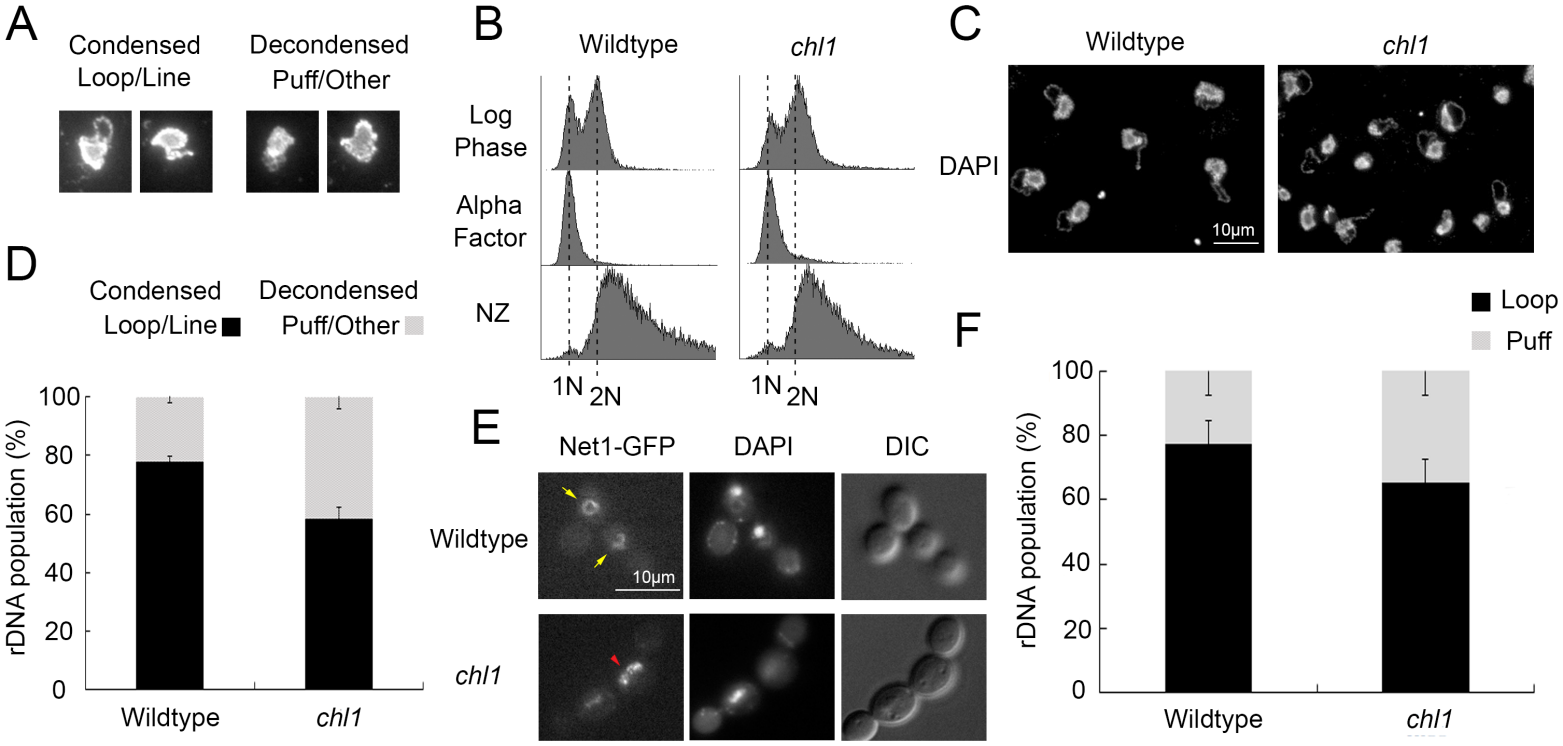
Chl1 helicase promotes rDNA condensation. A) Representative examples of micrographs that highlight condensed (loop and line) and decondensed (amorphous puff-like and other non-discrete configuration) rDNA structures. B) Flow cytometer of DNA content at times indicated throughout the experimental procedure. Cells were maintained in nocodazole for 3 hours at 23°C post-alpha factor arrest. C) Chromosome mass and rDNA detected using DAPI in wildtype (YBS1019) and *chl1* mutant (YBS1041) strains. D) Quantification of condensed (loop/line) and decondensed (puff/other) rDNA populations in wildtype and *chl1* mutant cells. Data quantified from 3 biological replicates, 100 cells for each strain analyzed per replicate and statistical analysis performed using Student's T-test (p = 0.005). E) rDNA structures visualized using Net1-GFP, genome DNA detected using DAPI, and cell morphology images obtained using Differential Interference Contrast (DIC) microscopy. Yellow arrows indicate condensed rDNA loop/line and red arrowhead indicates decondensed rDNA puff. F) Quantification of condensed (loop) and decondensed (puff) rDNA populations in wildtype (YBS2020) and *chl1* mutant (YBS2080) cells. Data quantified from 3 biological replicates, 100 cells for each strain analyzed per replicate and statistical analysis performed using Student's T-test (p = 0.006). Statistical significant differences (*) are based on p < 0.05.

Chl1 DNA helicase is critical for Scc2 recruitment to DNA [25], but the extent through which SMC-dependent DNA compaction is regulated through Chl1 remains untested. Here, we exploit the structural changes that rDNA undergoes across the cell cycle. In yeast, rDNA comprises up to 150 copies of linearly arrayed 9 kb sequence that form a diffuse and amorphous puff-like structure during G_1_ and condense into a discrete line and loop-like structure during mitosis [43], [44]. [47], [48]. Fluorescence in situ Hybridization (FISH) is a well-established methodology for detecting structural changes of rDNA loci, but is both time intensive and involves sequential application of three different antibodies and labeled probe [43], [44]. Previously, we developed and validated a streamlined condensation assay based on FISH but one that produces exquisite imaging of rDNA in the absence of antibodies and hybridization of labeled probe (Fig 1A) [45]. To assess the impact of Chl1 helicase on rDNA structure, wildtype and *chl1* deletion cells were synchronized in G_1_ using medium supplemented with alpha factor, washed and released into fresh medium supplemented with nocodazole for 3 hours. The resulting synchronized pre-anaphase cells were then processed to quantify the status of rDNA condensation (Fig 1B). The results confirm that wildtype cells exhibit high levels (78%) of tightly condensed (loop/line-like) rDNA loci while *chl1* mutant cells exhibit significantly lower levels (58%) of condensed rDNA loci. In fact, *chl1* mutant cells exhibited nearly twice the frequency of decondensed rDNA than wildtype cells (Fig 1C and 1D).

Chl1 DNA helicase is not essential for DNA replication, but we were nonetheless concerned that loss of Chl1 might produce a minor cell cycle delay that could be misinterpreted as a condensation defect. We assessed this possibility in three ways. First, we assessed cells after only 2.5 hours of preanaphase synchronization in medium supplemented with nocodazole. The results obtained by flow cytometry clearly reveal that both wildtype and *chl1* mutant cells are synchronized at this early step in the arrest protocol (S1 Fig). Thus, any imperceptible cell cycle delays will be fully compensated for by the additional 30 minutes of synchronization in the procedure described above. Second, we exploited the well-established changes in yeast cell morphology in which G_1_ cells are unbudded, S phase entry typically correlates with bud emergence, and subsequent G_2_ and M phases denoted by increased bud growth [49]. Our results reveal nearly identical large budded populations of wildtype and *chl1* mutant cells after 3 hours arrest in nocodazole (S1 Fig). Third, we quantified the minor 1N DNA peak in the preanaphase arrested cultures. The results reveal that 9.6% of wildtype cells exhibit a 1N DNA content while only 6.2% *chl1* mutant cells exhibit a 1N DNA content. Thus, *chl1* mutant cells arrest in preanaphase as efficiently as wildtype cells, negating the model that the two-fold increase in decondensed rDNA is due to cell cycle progression defects (S1 Fig).

Net1 is an rDNA binding protein such that Net1-GFP is another established method from which to monitor for architectural changes within the rDNA locus [23], [26], [42], [45], [50]. To independently assess for rDNA condensation defects in *chl1* mutant cells, Net1-GFP transformants of wildtype and *chl1* deletion cells were synchronized in pre-anaphase (S1 Fig), and the status of rDNA condensation (loops/lines versus puffs) quantified as previously described [23], [42–44]. As expected, wildtype cells exhibited high levels (77%) of condensed (loop/line-like) rDNA loci. In contrast, *chl1* mutant cells exhibited significantly lower levels (65%) of condensed rDNA loci and over a 50% increase in the number of rDNA puff structures, compared to wildtype cells (Fig 1E and 1F). Importantly, the impact of *chl1* mutation on rDNA structure occurs in the absence of shifting to elevated temperatures (37°C), a procedure that significantly impacts rDNA structure even in wildtype cells [45]. These combined results therefore reveal that Chl1 promotes condensation of the rDNA locus.

### Chl1 helicase promotes arm condensation

**Fig 2:**
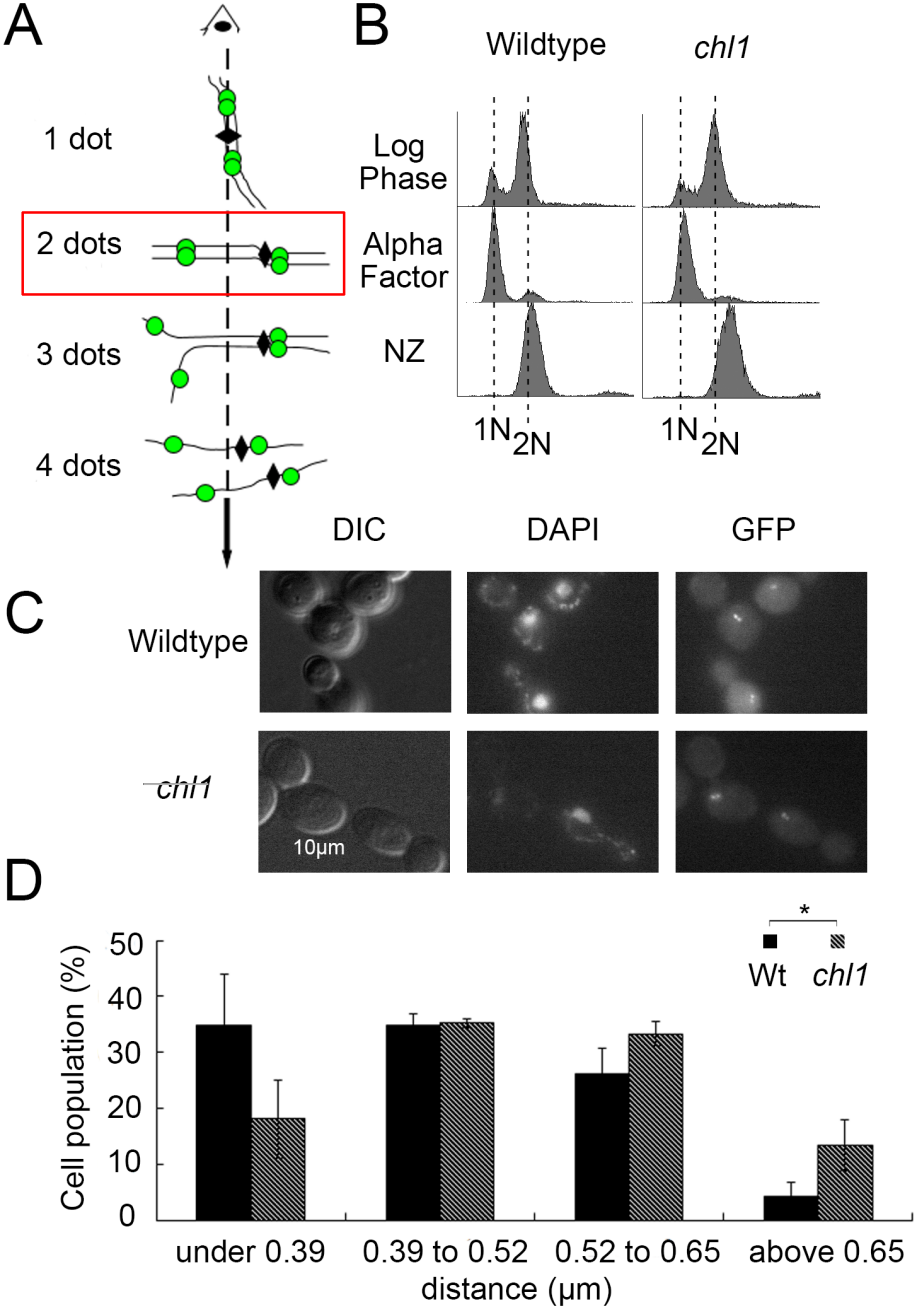
Chl1 helicase promotes chromosome arm condensation. A) Schematic of chromosome conformations and GFP-labeled loci (dashed line indicates Z-axial microscope orientation, solid line indicates sister chromatid, diamond indicates centromere, green dot indicate GFP locus). Red box indicates cells analyzed. B) Flow cytometer data of DNA content throughout the experiment. Cells were maintained in nocodazole for 3 hours at 23°C post-alpha factor arrest. C) Micrographs of representative fields of view that include GFP loci, genomic mass (DAPI) and cell morphology (DIC). D) Distribution of distances measured between GFP dots in wildtype (YBS2078) and *chl1* mutant (YBS2079) cells. Data obtained from 3 biological replicates, 100 cells for each strain analyzed per replicate and statistical analysis performed using Student's T-test. p = 3.862E-6 indicates the significant differences between the average distance (0.44 μm) in wildtype cells versus the average distance (0.50 μm) in *chl1* mutant cells. Statistical significant differences (*) are based on p < 0.05.

rDNA is unique in architecture, associated factors, transcription regulation, and recombination frequency compared to the remainder of the genome [51]. Thus, it became important to assess if Chl1 is an rDNA-specific regulator of condensation or instead impacts condensation genome-wide. We obtained from the lab of Dr. Frank Uhlmann a chromosome arm condensation assay strain that contains two lacO repeats, one integrated telomere-proximal on the left arm and another integrated centromere-proximal on the right arm of chromosome XII [23]. Each LacO cassette is detectable through lacI-GFP binding such that the inter-GFP distance allows for quantification of chromosome arm condensation (Fig 2A). Isogenic chromosome arm condensation assay strains, except for deletion of *CHL1*, were synchronized in G_1_ using alpha factor, washed and released into fresh medium supplemented with nocodazole (Fig 2B). The resulting pre-anaphase synchronized cells were then fixed in paraformaldehyde and the disposition of arm condensation quantified by measuring the distance between GFP loci. As expected, *chl1* mutant cells exhibit cohesion defects [27], [32] and we therefore encountered a range of detectable GFP loci. We thus limited our analysis to wildtype and *chl1* mutant cells that contained only 2 GFP dots and in which both were resolvable within a single focal plane (Fig 2A). We found a wide range of inter-GFP distances both in wildtype and *chl1* mutant cells. Regardless, the results reveal that 70% of wildtype cells exhibited inter-GFP distances under 0.52 μm. In contrast, only 53% of *chl1* mutant cells exhibited inter-GFP distances under 0.52 μm. In fact, *chl1* mutant cells exhibited inter-GFP distances above 0.65 μm at roughly 3 times the frequencies of wildtype cells (Fig 2C and 2D). These results reveal for the first time that Chl1 plays a genome-wide role in chromosome condensation.

### Chl1 and Scc2 function in condensation independent of condensin deposition

**Fig 3:**
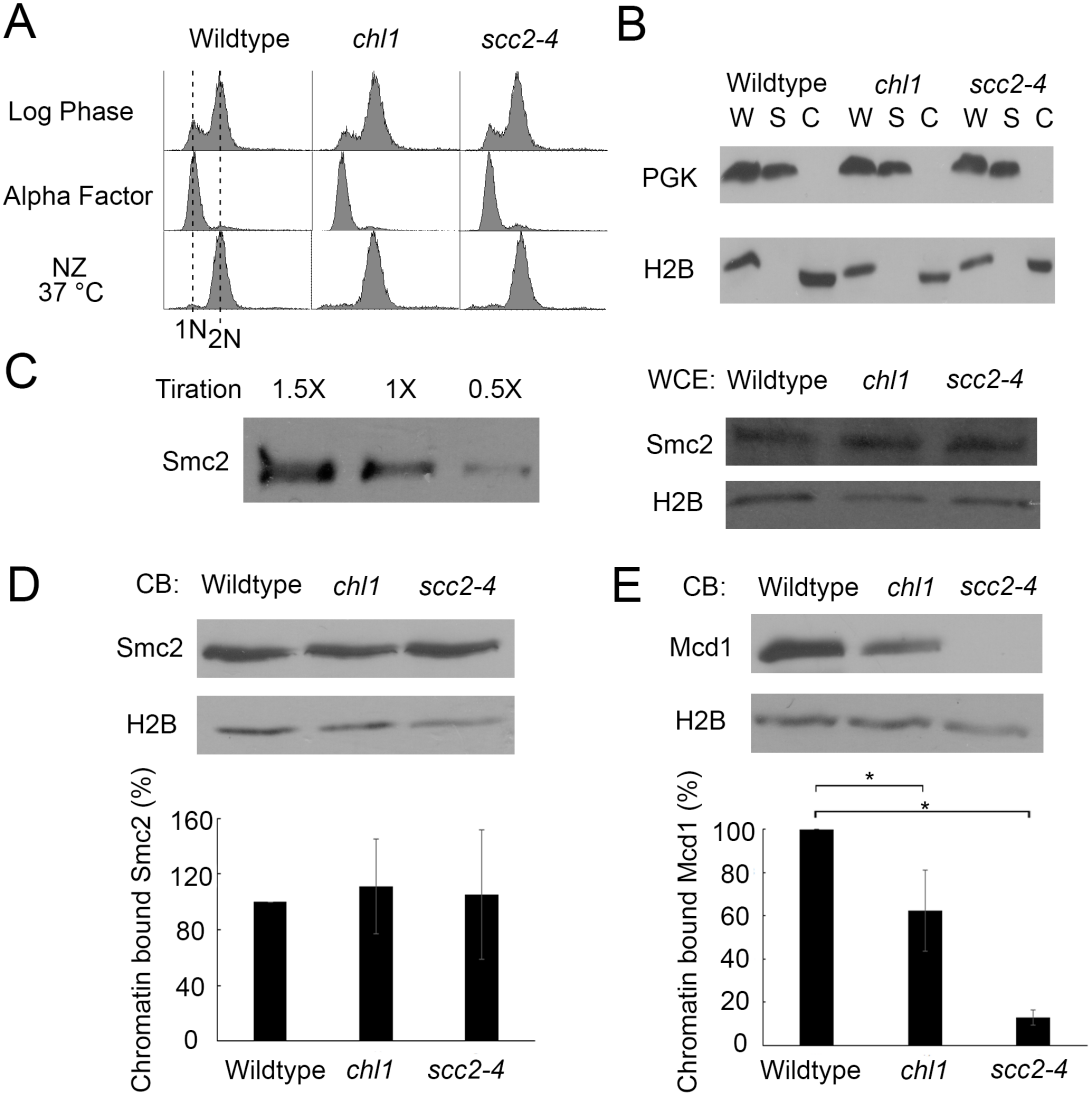
Chl1 helicase promotes chromosome condensation through cohesin, but not condensin, regulation. A) Flow cytometer data of DNA content throughout the experiment. Cells were maintained in nocodazole for 3 hours at 37°C post-alpha factor arrest. B) Fractionation of preanaphase-arrested wildtype (YDS101), *chl1* (YDS104) and *scc2-4* (YDS108) cells. Phosphoglycerate kinase (PGK) and Histone 2B (H2B) indicate levels of cytoplasmic and chromatin-bound proteins, respectively, in whole cell extracts (W), cytoplasmic soluble fractions (S) and chromatin bound fractions (C). C) Left: Titration of Smc2-HA indicates 1X sample concentration is in the linear range of detection. Right: Whole cell extracts of Smc2-HA in wildtype, *chl1* and *scc2-4* cells. H2B is shown as internal loading control. All samples reflect 1X concentration levels. D) Top: Chromatin-bound (CB) fraction of Smc2-HA in wildtype, *chl1* and *scc2-4* cells. Chromatin-bound H2B levels are shown as internal loading control. Bottom: Quantification of Smc2-HA binding to chromatin in *chl1* and *scc2-4* mutant cells, based on the ratio of Smc2-HA to H2B levels and normalized to wildtype levels of Smc2-HA obtained from 3 biological replicates. E) Top: Chromatin-bound fraction of Mcd1 in wildtype, *chl1* and *scc2-4* cells. Chromatin-bound H2B levels are shown as loading controls. Bottom: Quantification of Mcd1 binding to chromatin in *chl1* and *scc2-4* mutant cells, based on the ratio of Mcd1 to H2B levels and normalized to wildtype levels obtained from 3 biological replicates. Statistical analysis was performed using one-way ANOVA followed by post-hoc Tukey HSD Test. (p = 0.024 for chromatin bound Mcd1 in wildtype versus *chl1* mutant cells. p = 0.001 for chromatin bound Mcd1 in wildtype cells versus *scc2-4* mutant cells). Statistical significant differences (*) are based on p < 0.05.

What is the mechanism through which Chl1 and Scc2 function in condensation? Chl1 is well-documented as a cohesion regulator that is critical for Scc2 recruitment to chromatin [25]. In turn, Scc2 is essential for cohesin deposition onto chromatin, but a role for Scc2 in condensin deposition remains controversial [19], [23]. Thus, it became critical to differentiate between models that Chl1 promotes chromatin compaction through either Scc2-dependent regulation of condensins, cohesins, or both. We first tested whether the condensation defect produced in *chl1* mutant cells occurs through the reduction of condensin deposition onto DNA. Condensin subunit Smc2 was epitope-tagged as the sole source of Smc2 function in both wildtype and *chl1* mutant cells. We included *scc2-4* cells in our analyses so that we could directly compare the roles of Chl1 and Scc2 on condensin deposition. We then exploited Triton X-100 cell fractionation assays previously used to demonstrate chromatin-association of a spectrum of factors that include Ctf7/Eco1, cohesin subunits, DNA replication initiators and fork stabilization proteins [25], [52–57]. Wildtype, *chl1* and *scc2-4* single mutant strains each expressing Smc2-HA were synchronized in G_1_ (alpha factor), washed and released at 37°C (non-permissive for *scc2-4*) into fresh medium supplemented with nocodazole (Fig 3A). We validated the cell fractionation procedure using Phosphoglycerokinase (PGK) and Histone 2B (H2B) as cytosolic and chromatin fiduciary markers, respectively. The results show efficient enrichment of H2B, and undetectable levels of PGK, in Triton-X-100 insoluble chromatin bound fractions (Fig 3B). Smc2 titration demonstrates that protein loading is within the linear range of detection (Fig 3B). We first compared the total levels of Smc2-HA, normalized to H2B levels, in whole cell extractions obtained from wildtype, *chl1* and *scc2-4* single mutant cells. The results from whole cell extracts document that Smc2-HA levels are unaffected in either *chl1* or *scc2-4* mutations, compared to wildtype cells (Fig 3C). We then compared the levels of chromatin-bound Smc2-HA. The result revealed that chromatin-bound Smc2-HA levels in both *chl1* and *scc2-4* mutant cells are not reduced, compared to wildtype cells (Fig 3D). Thus, *chl1* and *scc2-4* single mutant cells exhibit significant condensation defects despite full retention of chromatin-bound condensin. These results document that the condensation defects exhibited by *chl1* and *scc2-4* single mutant cells occur largely independent of changes in condensin deposition onto DNA.

### Chl1 and Scc2 function in condensation through cohesins

Having eliminated reduced condensin deposition as a central mechanism through which *chl1* and *scc2-4* mutants produce condensation defects, we turned to cohesin deposition. Wildtype, *chl1* and *scc2-4* single mutant strains were released from G_1_ into 37°C medium supplemented with nocodazole and the resulting preanaphase cells assayed for cohesin deposition. As before, we confirmed the Triton X-100 cell fractionation assay using PGK and H2B as cytosolic and chromatin fiduciary markers, respectively (Fig 3B). We and others previously ascertained that Mcd1 levels in whole cell lysates are unaffected in either *chl1* or *scc2-4* mutant cells [19], [25]. Thus, we compared the levels of chromatin bound Mcd1, normalized to H2B levels, in wildtype and *chl1* and *scc2-4* single mutant cells using a previously validated Mcd1/Scc1-directed antibody generously provided by Dr. Vincent Guacci of the Koshland Lab [44]. As expected from prior studies, Mcd1 binding to DNA to significantly reduced in *chl1* (38%) and *scc2-4* (82%) single mutant cells (Fig 3E). Notably, the reductions in Mcd1 binding mirrored the severity of the condensation defect, strongly indicative of a dose-dependent cohesin mechanism (Fig 3E). Independently, we repeated the assessment of Smc2-HA in these chromatin fractions now validated for scc2-4 inactivation through reduced cohesin levels. Our results confirm that Scc2 inactivation has negligible effects on condensin deposition.

**Fig 4:**
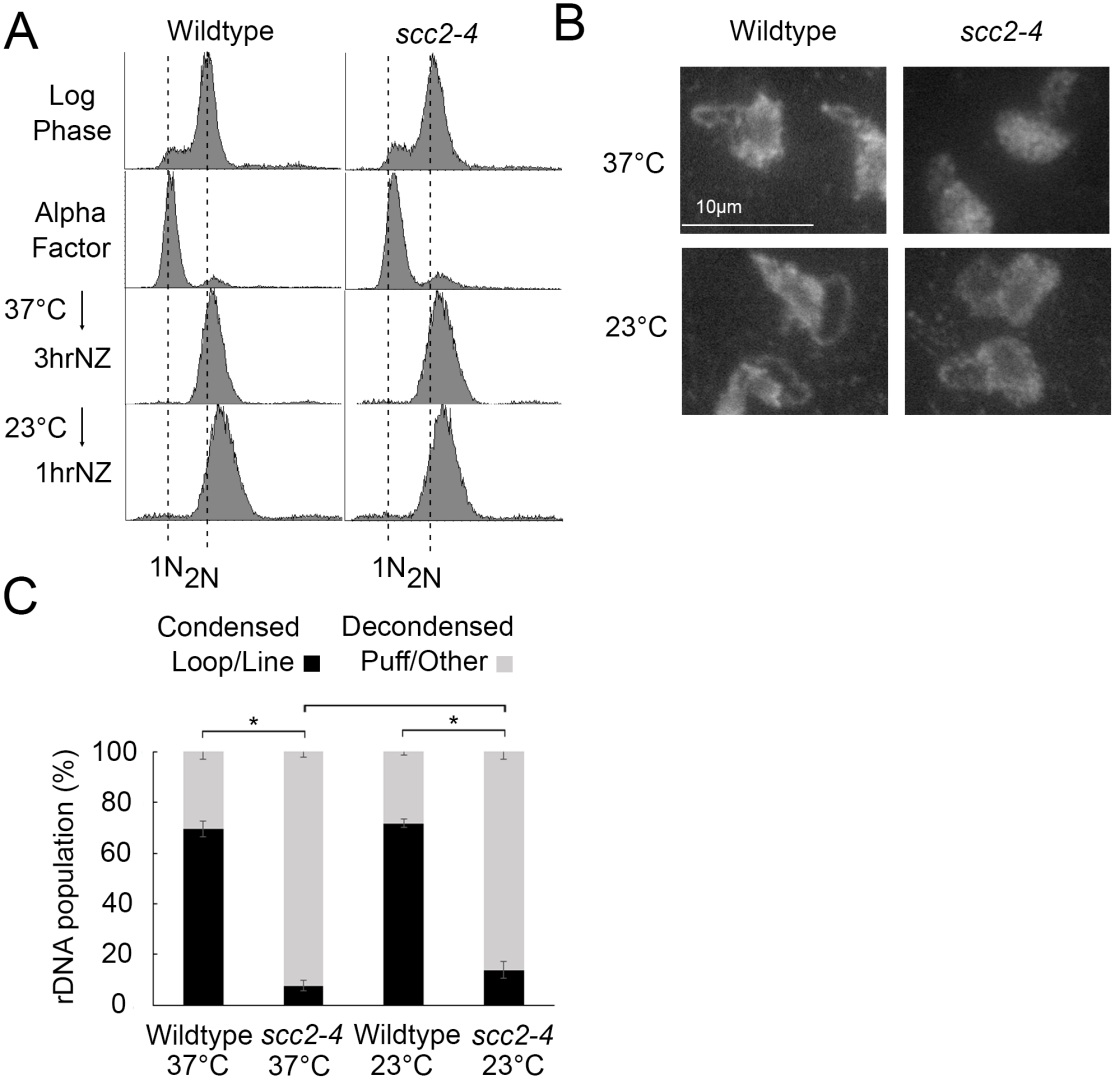
Condensation is irreversible in *scc2-4* mutants. A) Flow cytometer data reveals DNA content throughout the experimental analyses. Cells were maintained in nocodazole, post-alpha factor release, for 3 hours at 37°C followed by an additional 1 hour at 23°C. B) Chromosome mass and rDNA structures detected using DAPI in wildtype and *scc2-4* mutant strains. C) Quantification of condensed (loop/line) and decondensed (puff/other) rDNA populations in wildtype (YBS1019) and *scc2-4* mutant (YMM551) cells. Quantifications and statistical analyses of rDNA condensation were obtained from 3 biological replicates for each strain (wildtype and *scc2-4* mutant cells) in which each replicate included 100 cells for each strain. Statistical analyses of condensed populations were performed using one-way ANOVA followed by post-hoc Tukey HSD Test (p = 0.001 for wildtype versus *scc2-4* mutant cell rDNA condensation at 37°C; p = 0.001 for wildtype versus *scc2-4* mutant cell rDNA condensation at 23°C; p = 0.890 for wildtype cell rDNA condensation at 37°C versus 23°C; p = 0.301 for *scc2-4* mutant cell rDNA condensation at 37°C versus 23°C). Statistical significant differences (*) are based on p < 0.05.

While rDNA condensation defects are completely reversible in condensin mutant cells, condensation defects are irreversible in cohesin mutants [58]. If Scc2 promotes condensation only through cohesin deposition, then condensation should be irreversible in *scc2-4* mutant cells. To test this prediction, wildtype and *scc2-4* mutant strains were released from G_1_ into 37°C medium supplemented with nocodazole for 3 hours and the resulting preanaphase cultures then shifted back to 23°C for 1 hour (Fig 4A). Cell samples harvested both during the preanaphase arrest at 37°C and after the shift down to 23°C were then assessed for the disposition of rDNA condensation as previously described [45]. As expected, preanaphase wildtype cells arrested at 37°C exhibited high levels (69%) of condensed (loop-like) rDNA loci while *scc2-4* mutant cells instead exhibited significantly low levels (8%) of condensed rDNA loci (Fig 4B). Upon shifting down to 23°C, preanaphase wildtype cells continued to exhibit high levels (71%) of condensed (loop-like) rDNA loci. Importantly, *scc2-4* mutant cells also exhibited significantly low levels (14%) of condensed rDNA loci (Fig 4C). The predominantly irreversible condensation defect in *scc2-4* mutant cells mirrors that of cohesin mutants and is distinct from the complete rescue of condensation defects exhibited in condensin mutant cells following the same regimen [58]. The combination of these results strongly suggest that the condensation defects exhibited by *chl1* and *scc2-4* mutant cells occur predominantly through a reduction in cohesin, but not condensin, chromatin association.

### Scc2 plays a mitotic role in arm condensation but not rDNA condensation

**Fig 5:**
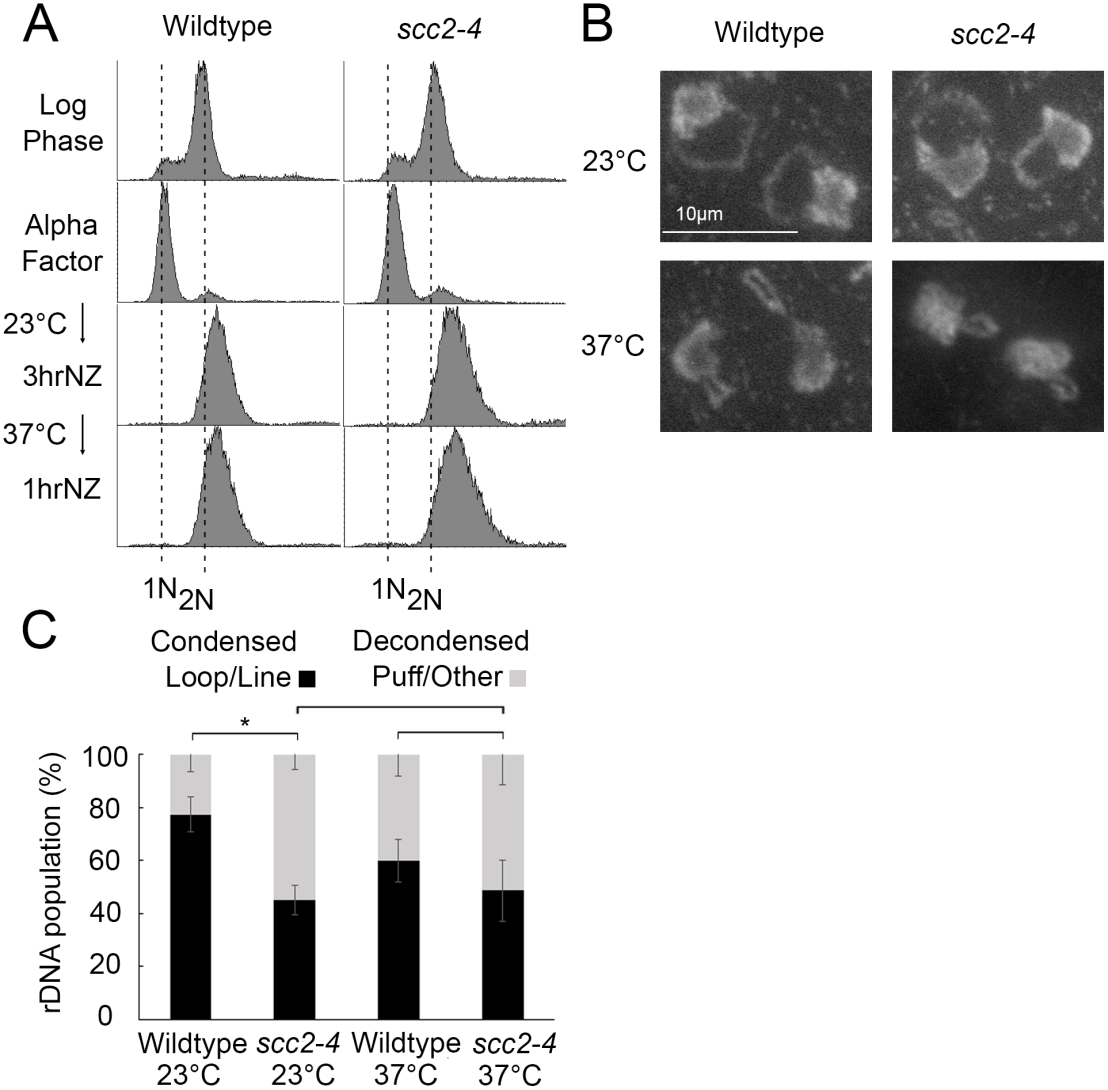
Scc2 is dispensable for condensation maintenance during M phase. A) Flow cytometer data reveals DNA content throughout the experimental analyses. Cells were maintained in nocodazole, post-alpha factor release, for 3 hours at 23°C followed by an additional 1 hour at 37°C. B) Chromosome mass and rDNA structures detected using DAPI in wildtype and *scc2-4* mutant strains. C) Quantification of condensed (loop/line) and decondensed (puff/other) rDNA populations in wildtype (YBS1019) and *scc2-4* mutant (YMM551) cells. Quantifications and statistical analyses of rDNA condensation were obtained from 3 biological replicates for each strain (wildtype and *scc2-4* mutant cells) in which each replicate included 100 cells for each strain. Statistical analyses of condensed populations were performed using one-way ANOVA followed by post-hoc Tukey HSD Test (p = 0.014 for wildtype cell versus *scc2-4* mutant cell rDNA condensation at 23°C; p = 0.804 for wildtype cell versus *scc2-4* mutant cell rDNA condensation at 37°C; p = 0.133 for wildtype cell rDNA condensation at 23°C versus 37°C; p = 0.878 for *scc2-4* mutant cell rDNA condensation at 23°C versus 37°C). Statistical significant differences (*) are based on p < 0.05.

Scc2 inactivation during S phase causes severe chromosome condensation defects and cell lethality [19]. Intriguingly, there is limited evidence that Scc2 inactivation specifically during M phase also produces chromosome arm condensation defects, even while cells retain high viability [23]. We were intrigued by the possibility that *scc2-4* cell viability, during M phase inactivation, might be explained if rDNA remains condensed even while chromosome arms decondense. To test this possibility, wildtype and *scc2-4* mutant strains were synchronized in G_1_ in medium supplemented with alpha factor, released into 23°C medium supplemented with nocodazole for 3 hours, and the resulting preanaphase cultures then shifted to 37°C for 1 hour to inactivate *scc2-4* specific during M phase (Fig 5A). Cell samples were processed to determine the status of rDNA condensation as previously described [45]. As expected, preanaphase wildtype cells maintained at 23°C exhibited high levels (77%) of condensed (loop-like) rDNA loci. Even at this temperature permissive for cell viability, *scc2-4* mutant cells exhibited surprisingly modest levels (45%) of condensed rDNA loci (Fig 5B and 5C). Preanaphase wildtype cells shifted to 37°C retained high levels (60%) of condensed rDNA, albeit with shorter rDNA loops as previously described [45]. Importantly, preanaphase *scc2-4* mutant cells shifted to 37°C similarly retained its modest level of condensed rDNA (48%) loci (Fig 5B and 5C). These results reveal that Scc2 is not required for condensation maintenance of rDNA during M phase, in contrast to the role played by Scc2 in condensation along chromosome arms [23]. Thus, Scc2-dependent cohesin roles in condensation differentially effects rDNA and chromosome arm loci during mitosis.

## Discussion

Analyses of Chl1 helicase family members are of immediate clinical relevance. Mutations in *CHL1* human homologs BACH1/BRIP/FANCJ and ChlR1/DDX11 helicases collectively result in Warsaw Breakage Syndrome, Fanconi anemia, breast and ovarian cancers [27], [35–37], [40], [59–61]. A link between the Chl1 helicase family and global changes in chromatin structure, however, remained untested. The first major revelation of the current study is that Chl1 is an important factor in promoting genome-wide chromosome condensation. Intriguingly, we found that *chl1* mutants exhibit both chromosome arm and rDNA condensation defects, but at relatively moderate levels. This suggests that additional factors may support Chl1 in condensation reactions (including Scc2 and cohesin deposition onto DNA) and that cohesin-dependent condensation is taken place in S phase. Regardless, our findings extend the role of Chl1 beyond *trans* tethering (required for sister chromatid cohesion and DNA repair) to now include *cis* tethering [27], [31–34]. This distinction is critical in that *cis* tethering during G_1_ is thought to stabilize intramolecular DNA loops through which regulatory elements (enhancers, promoters, insulators) are brought into registration and thus deploy developmental transcription programs [1], [2]. Moreover, *cis* tethering also mediates both regional and genome-wide compaction reactions throughout the cell cycle—the latter of which is required for chromosome segregation. While the current study is unique in identifying a role for Chl1 DNA helicase in chromatin condensation, we note that mutation of other helicases (*MCM7*, *MCM10*, *FBH1*) in *C*. *elegans* or *Drosophila* models produced chromosome condensation defects, but effects attributed to incomplete replication and often with minimal effects on chromosome segregation [62–66]. Our findings suggest a reevaluation of current models may be warranted.

How does Chl1 DNA helicase promote DNA condensation? A second revelation of the current study is that Chl1, and its downstream target Scc2, function predominantly through cohesin-based condensation. In the current study, we simultaneously monitored both cohesin and condensin chromatin binding levels and found that only cohesins are adversely affected in *chl1* and *scc2-4* mutant strains. Intriguingly, a prior study found that *scc2-4* inactivation during a mitotic-arrest produced a modest condensin binding defect, but an effect predicated on perceived changes in fluorescent intensity levels obtained from chromosome spreads. Notably, that study provided inconsistent data regarding reductions in condensin recruitment to loci assessed by chromatin immunoprecipitations [23]. While our current findings diminish the role of Scc2 (and Chl1) in condensin recruitment, we note that mutation of the RNA helicase *Vasa*, which produce condensation defects in mitotic germ-line *Drosophila* cells, exhibit reduced recruitment of the condensin SMC capping factor Barren to DNA [67]. Thus, it will be critical to elucidate the extent through which different model cell systems prioritize the use of SMC complexes to drive chromatin compaction. Assessing these possibilities is complicated, however, due to evidence that condensin recruitment may be mediated indirectly through reduced cohesin recruitment [58].

**Fig 6:**
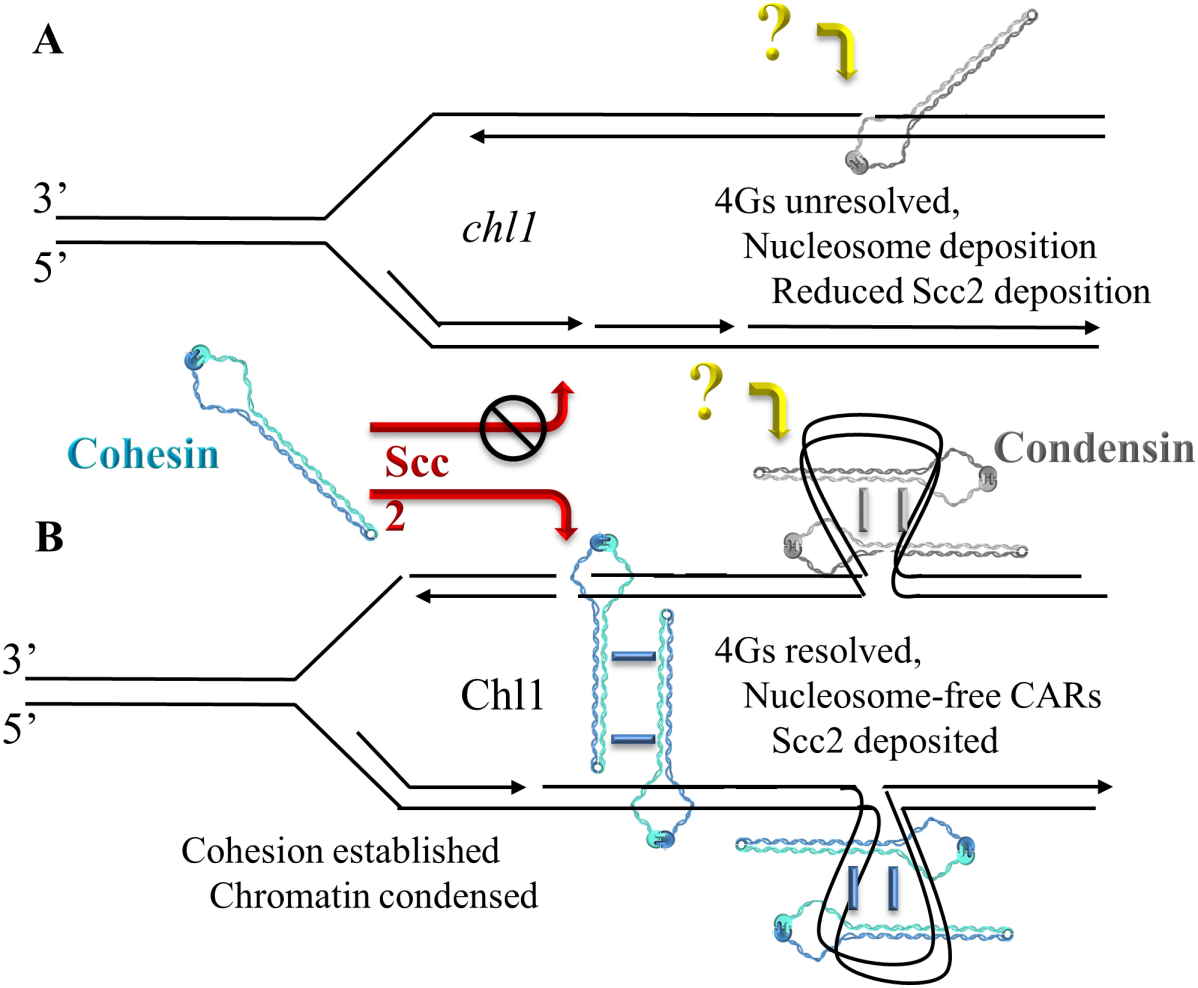
Chl1 DNA helicase functions in condensation. A) In the absence of Chl1, condensation defects occur despite normal recruitment of condensin (‘?’ reflects that condensin deposition, but not cohesin deposition, occurs despite Scc2 inactivation). We hypothesize that secondary DNA structures (such as G4s and nucleosomes) reduce both Scc2 and cohesin recruitment, resulting in cohesion and condensation defects. B) Chl1 activities (resolution of DNA secondary structures, histone displacement, etc) provides for both Scc2 and cohesin recruitment, resulting in sister chromatid cohesion (*trans* tethering) and chromatin condensation (*cis* tethering). Cohesin and condensin oligomerization are shown as one of several possible mechanisms of cohesion and condensation [1], [90].

Elucidating the mechanism through which Chl1 promotes both Scc2 and cohesin recruitment to DNA to mediate cohesion (*trans* tethering) and condensation (*cis* tethering) remains an important issue in cell biology. Elegant biochemical findings reveal that Chl1 family members resolve secondary DNA structures such as G4s, triple helices, and 5’ forked/flapped duplexes thought to arise either immediately behind the DNA replication fork or occur within specific loci throughout the genome [28], [34]. [68–74]. That these secondary DNA structures can be resolved in a post-fork context is strongly supported by findings that both Chl1 expression and chromatin binding peak during S phase and that Chl1 binds to numerous replication factors (such as Ctf4, Eco1, Fen1, Ctf18 and PCNA) that act in in conjunction with or immediately behind DNA polymerase [24], [27], [28], [30], [52], [54]. More recent findings posit that Scc2,4 binds DNA to maintain nucleosome-free domains onto which cohesins are later deposited [75]. Based on evidence that Chl1 family members disrupt streptavidin binding to biotinylated single-strand DNA oligonucleotides *in vitro* and resolve DNA secondary structures such as G quadruplexes (G4s) and triple DNA helices [70], [73], [76], we posit that Chl1 helicase actively promotes nucleosome-free domains that promote Scc2 and subsequent cohesin deposition (Fig 6). This helicase-based model in which DNA structure modulates Scc2 recruitment may equally apply to protein-based adaptors of Scc2,4 that include the elongation factor Paf1, Mediator transcription scaffold complex, the pre-Replication Complex (pre-RC), Ctf19/COMA kinetochore complex [77–89].

## Supporting information

S1 Fig Cell cycle progression in wildtype and *chl1* mutant. A) Morphology quantification for both nocodazole-arrested wildtype and *chl1* mutant cells (N = 100 cells for each strain). B) 1N peak quantification for both wildtype and *chl1* mutant cells arrested in nocodazole for 3 hours at 23°C. C) Flow cytometer data reveals DNA contents in wildtype and *chl1* mutant cells after nocodazole arrest at 23°C for 2.5 hours and 3 hours. D) Flow cytometer data reveals DNA content in wildtype and *chl1* mutant cells analyzed in Fig 1E and 1F.
(TIF)
