# Correction: Chl1 DNA helicase and Scc2 function in chromosome condensation through cohesin deposition

**DOI:** 10.1371/journal.pone.0227443

**Published:** 2020-01-10

**Authors:** Donglai Shen, Robert V. Skibbens

The authors would like to provide clarifications and correct errors in Figs 1, 3, 4 and S1 of the published article [1].

**Fig 1:**
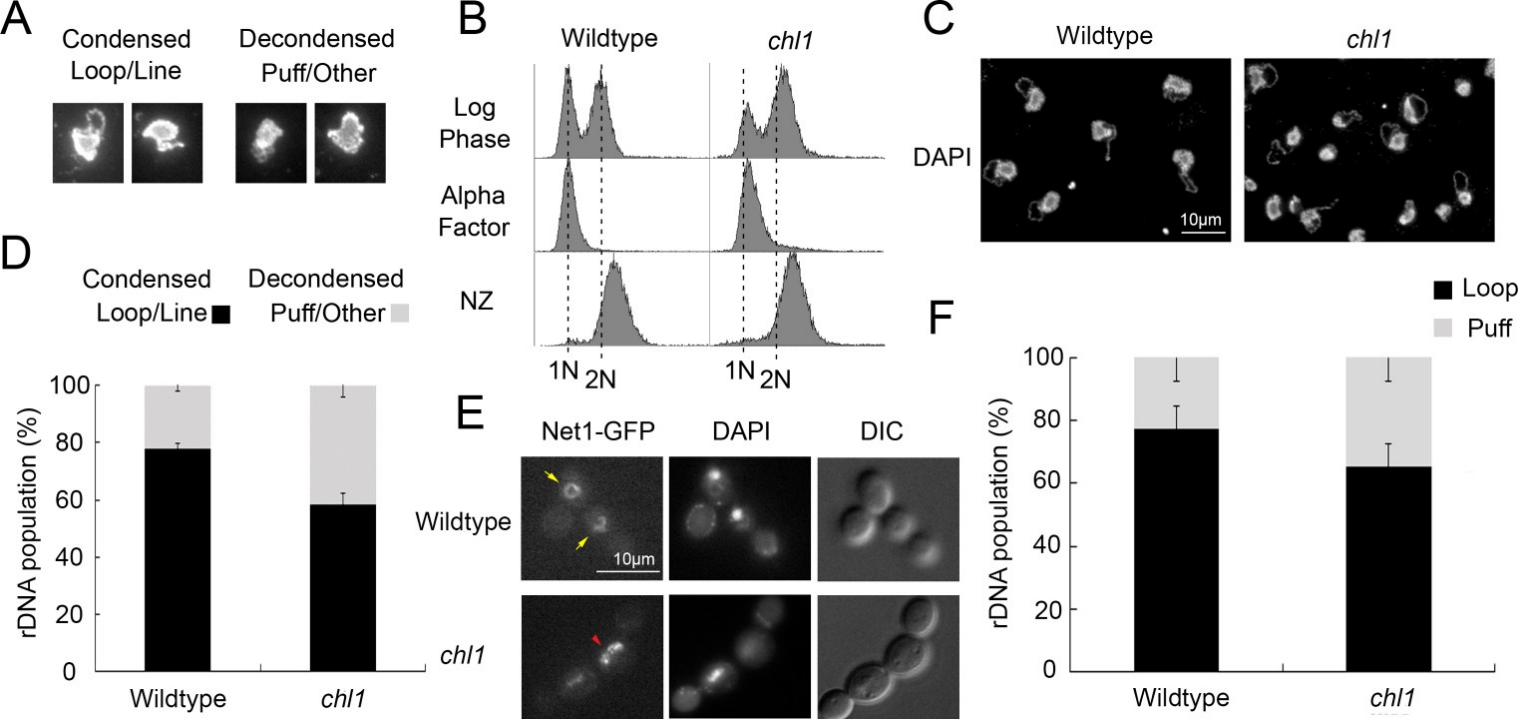
Chl1 helicase promotes rDNA condensation. A) Representative examples of micrographs that highlight condensed (loop and line) and decondensed (amorphous puff-like and other non-discrete configuration) rDNA structures. B) Flow cytometer of DNA content at times indicated throughout the experimental procedure. Cells were maintained in nocodazole for 3 hours at 23°C post-alpha factor arrest. C) Chromosome mass and rDNA detected using DAPI in wildtype (YBS1019) and *chl1* mutant (YBS1041) strains. D) Quantification of condensed (loop/line) and decondensed (puff/other) rDNA populations in wildtype and *chl1* mutant cells. Data quantified from 3 biological replicates, 100 cells for each strain analyzed per replicate and statistical analysis performed using Student's T-test (p = 0.005). E) rDNA structures visualized using Net1-GFP, genome DNA detected using DAPI, and cell morphology images obtained using Differential Interference Contrast (DIC) microscopy. Yellow arrows indicate condensed rDNA loop/line and red arrowhead indicates decondensed rDNA puff. F) Quantification of condensed (loop) and decondensed (puff) rDNA populations in wildtype (YBS2020) and *chl1* mutant (YBS2080) cells. Data quantified from 3 biological replicates, 100 cells for each strain analyzed per replicate and statistical analysis performed using Student's T-test (p = 0.006). Statistically significant differences (*) are based on p < 0.05.

**Fig 3:**
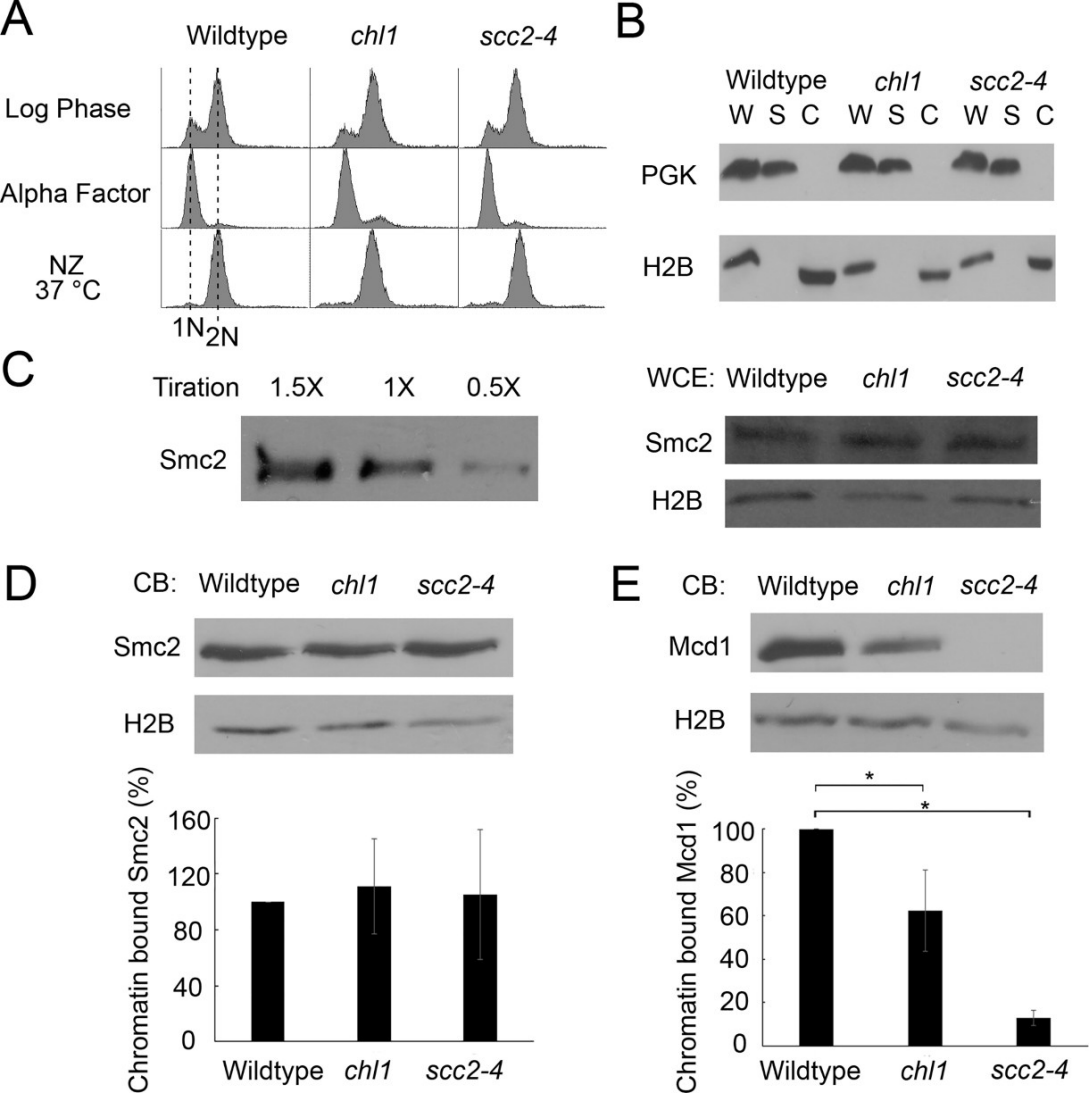
Chl1 helicase promotes chromosome condensation through cohesin, but not condensin, regulation. A) Flow cytometer data of DNA content throughout the experiment. Cells were maintained in nocodazole for 3 hours at 37°C post-alpha factor arrest. B) Fractionation of preanaphase-arrested wildtype (YDS101), *chl1* (YDS104) and *scc2-4* (YDS108) cells. Phosphoglycerate kinase (PGK) and Histone 2B (H2B) indicate levels of cytoplasmic and chromatin-bound proteins, respectively, in whole cell extracts (W), cytoplasmic soluble fractions (S) and chromatin bound fractions (C). C) Left: Titration of Smc2-HA indicates 1X sample concentration is in the linear range of detection. Right: Whole cell extracts of Smc2-HA in wildtype, *chl1* and *scc2-4* cells. H2B is shown as internal loading control. All samples reflect 1X concentration levels. D) Top: Chromatin-bound (CB) fraction of Smc2-HA in wildtype, *chl1* and *scc2-4* cells. Chromatin-bound H2B levels are shown as internal loading control. Bottom: Quantification of Smc2-HA binding to chromatin in *chl1* and *scc2-4* mutant cells, based on the ratio of Smc2-HA to H2B levels and normalized to wildtype levels of Smc2-HA obtained from 3 biological replicates. E) Top: Chromatin-bound fraction of Mcd1 in wildtype, *chl1* and *scc2-4* cells. Chromatin-bound H2B levels are shown as loading controls. Bottom: Quantification of Mcd1 binding to chromatin in *chl1* and *scc2-4* mutant cells, based on the ratio of Mcd1 to H2B levels and normalized to wildtype levels obtained from 3 biological replicates. Statistical analysis was performed using one-way ANOVA followed by post-hoc Tukey HSD Test. (p = 0.024 for chromatin bound Mcd1 in wildtype versus *chl1* mutant cells. p = 0.001 for chromatin bound Mcd1 in wildtype cells versus *scc2-4* mutant cells). Statistically significant differences (*) are based on p < 0.05.

**Fig 4:**
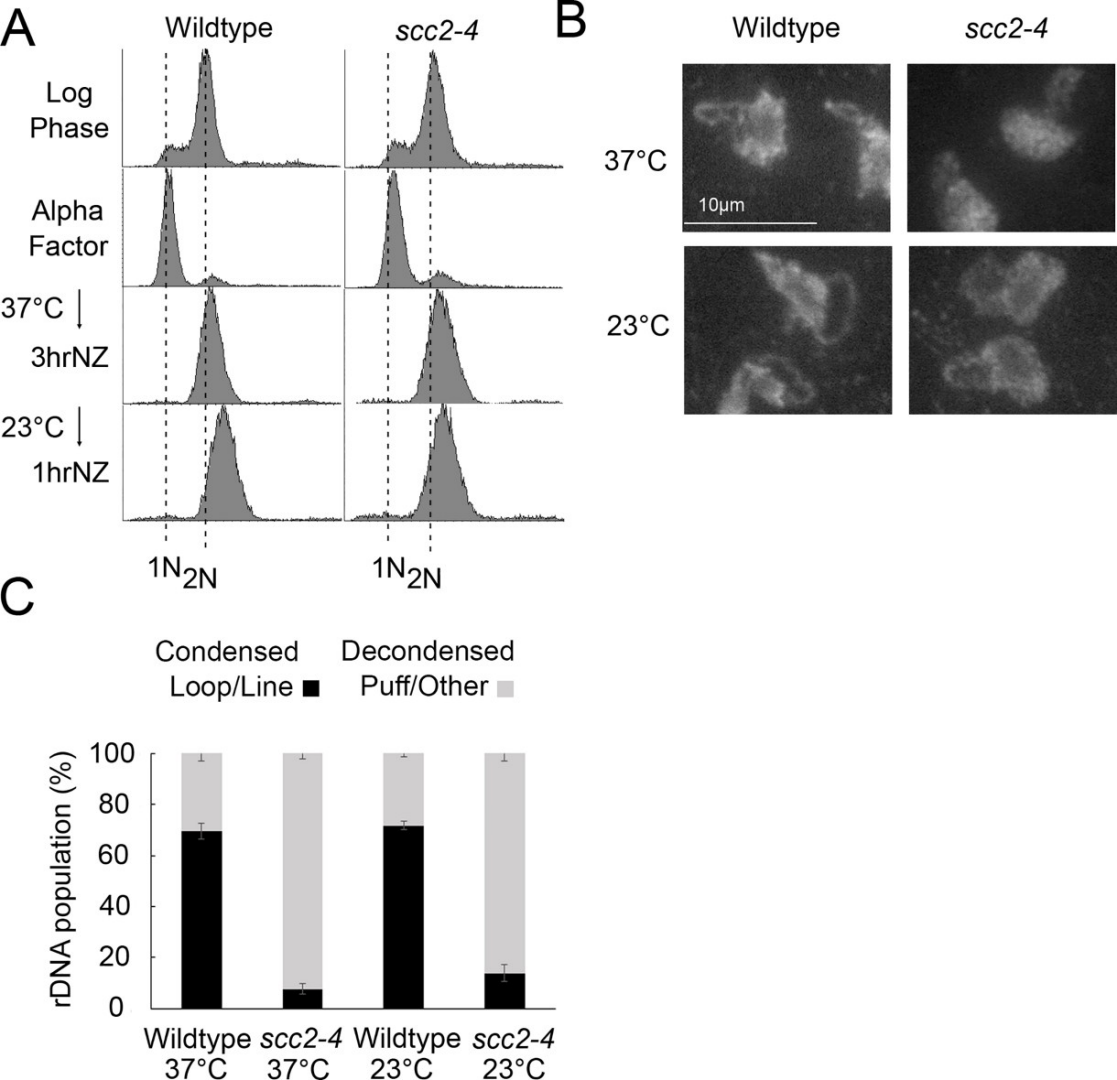
Condensation is irreversible in *scc2-4* mutants. A) Flow cytometer data reveals DNA content throughout the experimental analyses. Cells were maintained in nocodazole, post-alpha factor release, for 3 hours at 37°C followed by an additional 1 hour at 23°C. Log phase wildtype and *scc2-4* mutant cells, arrested and then released from a G1 arrest, were used in experiments depicted in both Fig 4 and Fig 5, as such the DNA profiles shown for Log phase and Alpha factor are the same in these two figures. B) Chromosome mass and rDNA structures detected using DAPI in wildtype and *scc2-4* mutant strains. C) Quantification of condensed (loop/line) and decondensed (puff/other) rDNA populations in wildtype (YBS1039) and *scc2-4* mutant (YMM551) cells. Quantifications and statistical analyses of rDNA condensation were obtained from 3 biological replicates for each strain (wildtype and *scc2-4* mutant cells) in which each replicate included 100 cells for each strain. Statistical analyses of condensed populations were performed using one-way ANOVA followed by post-hoc Tukey HSD Test (p = 0.001 for wildtype versus *scc2-4* mutant cell rDNA condensation at 37°C; p = 0.001 for wildtype versus *scc2-4* mutant cell rDNA condensation at 23°C; p = 0.890 for wildtype cell rDNA condensation at 37°C versus 23°C; p = 0.301 for *scc2-4* mutant cell rDNA condensation at 37°C versus 23°C). Statistically significant differences (*) are based on p < 0.05.

**Fig 5:**
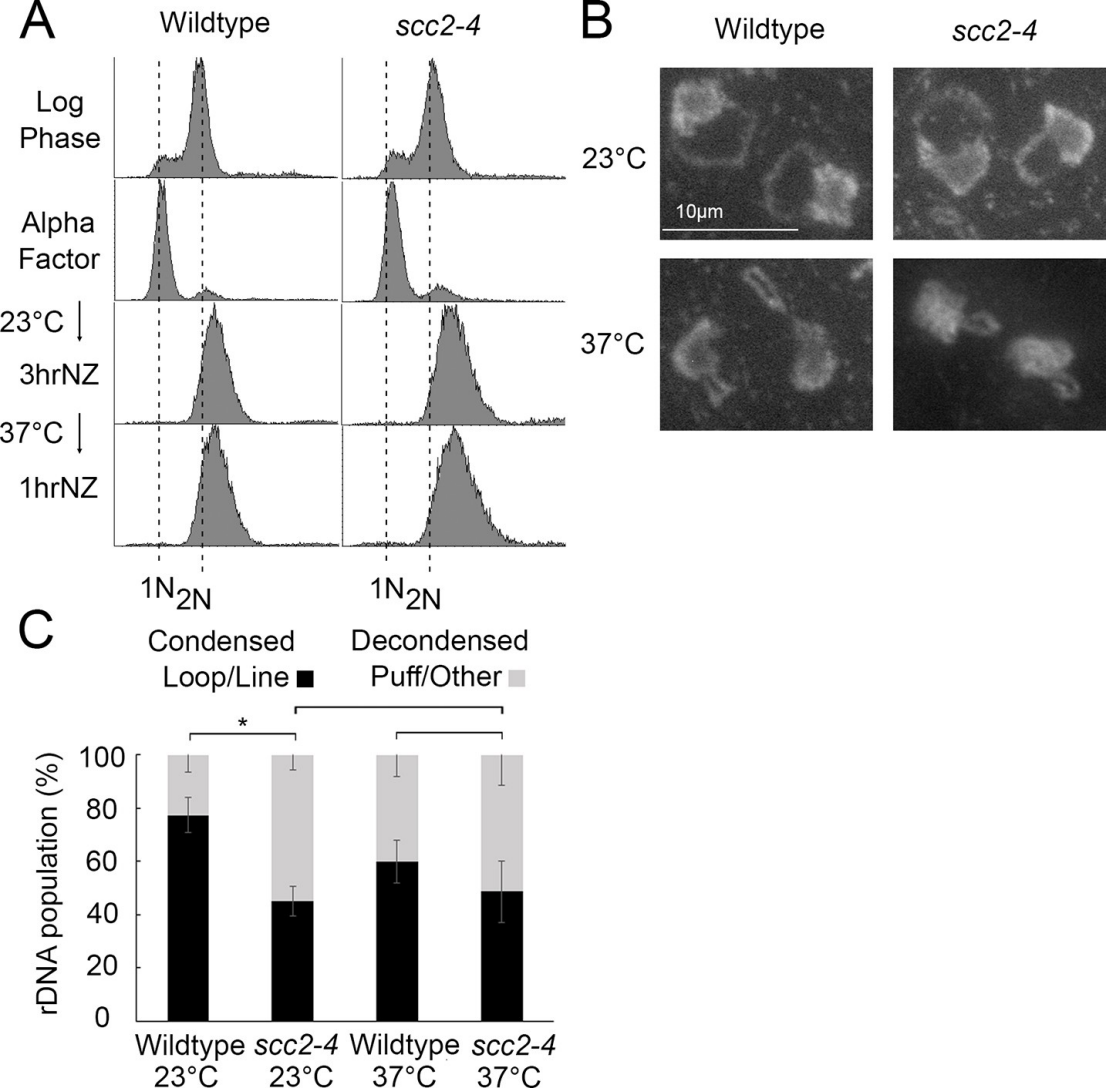
Scc2 is dispensable for condensation maintenance during M phase. A) Flow cytometer data reveals DNA content throughout the experimental analyses. Cells were maintained in nocodazole, post-alpha factor release, for 3 hours at 23°C followed by an additional 1 hour at 37°C. Log phase wildtype and scc2-4 mutant cells, arrested and then released from a G1 arrest, were used in experiments depicted in both Fig 4 and Fig 5, as such the DNA profiles shown for Log phase and Alpha factor are the same in these two figures. B) Chromosome mass and rDNA structures detected using DAPI in wildtype and *scc2-4* mutant strains. C) Quantification of condensed (loop/line) and decondensed (puff/other) rDNA populations in wildtype (YBS1039) and *scc2-4* mutant (YMM551) cells. Quantifications and statistical analyses of rDNA condensation were obtained from 3 biological replicates for each strain (wildtype and *scc2-4* mutant cells) in which each replicate included 100 cells for each strain. Statistical analyses of condensed populations were performed using one-way ANOVA followed by post-hoc Tukey HSD Test (p = 0.014 for wildtype cell versus *scc2-4* mutant cell rDNA condensation at 23°C; p = 0.804 for wildtype cell versus *scc2-4* mutant cell rDNA condensation at 37°C; p = 0.133 for wildtype cell rDNA condensation at 23°C versus 37°C; p = 0.878 for *scc2-4* mutant cell rDNA condensation at 23°C versus 37°C). Statistically significant differences (*) are based on p < 0.05.

The wildtype flow cytometry DNA panels in Fig 1B were previously published in S1B Fig of another article [2]. The authors confirm that original images used are accurate representative examples and belong to the dataset analysed for the *PLOS ONE* study, as both studies used the same repository of cells and treatments, and therefore some data was re-used. A revised Fig 1 is provided here, using replacement images for all panels of Fig 1B from alternative replicates of the same study.

The *PLOS ONE* study [1] was carried out using the raw images generated during the previous study [2]; however, the authors confirm the analyses reported in [1] are novel and unique.

In Fig 3, a duplicate of the Alpha factor DNA profile for wildtype cells was incorrectly used for the Alpha factor panel for *chl1* mutant cells. The incorrect panel is replaced in the revised Fig 3 provided here.

In Fig 4, a duplicate of the 3hr DNA profile for *scc2-4* cells was incorrectly used in the NZ 1hr panel for wildtype cells. The incorrect panel is replaced in the revised Fig 4 provided here.

In S1D Fig, a duplicate of the log phase DNA profile for wildtype cells was incorrectly used in the panel for log phase *chl1* mutant cells. The incorrect panel is replaced in the revised S1 Fig provided here.

The authors confirm that the above errors occurred in figure assembly and do not affect the analyses or findings of the study. The authors apologize for the errors.

The legends for Fig 4 and Fig 5 are updated to clarify that the Log phase and Alpha factor DNA profiles in Figs 4A and 5A are the same. Additionally, the wildtype yeast strain was incorrectly named YBS1019; this is corrected to YBS1039 in both figure legends. Please see the complete, correct Fig 5 caption here.

The underlying data for the article is provided on Figshare: https://doi.org/10.6084/m9.figshare.11412783.v2.

## Supporting information

S1 Fig Cell cycle progression in wildtype and *chl1* mutant. A) Morphology quantification for both nocodazole-arrested wildtype and *chl1* mutant cells (N = 100 cells for each strain). B) 1N peak quantification for both wildtype and *chl1* mutant cells arrested in nocodazole for 3 hours at 23°C. C) Flow cytometer data reveals DNA contents in wildtype and *chl1* mutant cells after nocodazole arrest at 23°C for 2.5 hours and 3 hours. D) Flow cytometer data reveals DNA content in wildtype and *chl1* mutant cells analyzed in Fig 1E and 1F.
(TIF)

## References

[1] Shen D, Skibbens RV (2017) Chl1 DNA helicase and Scc2 function in chromosome condensation through cohesin deposition. PLoS ONE 12(11): e0188739 10.1371/journal.pone.0188739 29186203 PMC5706694

[2] Shen D, Skibbens RV (2017) Temperature-dependent regulation of rDNA condensation in Saccharomyces cerevisiae. Cell Cycle 16(11): Pages 1118–1127 10.1080/15384101.2017.1317409 28426272 PMC5499843

