# Supplementary figures and images for: Correction: Chl1 DNA helicase and Scc2 function in chromosome condensation through cohesin deposition
Source: PLoS One. 2020 Jan 10;15(1):e0227443. doi: 10.1371/journal.pone.0227443 (PMC6953843; doi:10.1371/journal.pone.0227443)

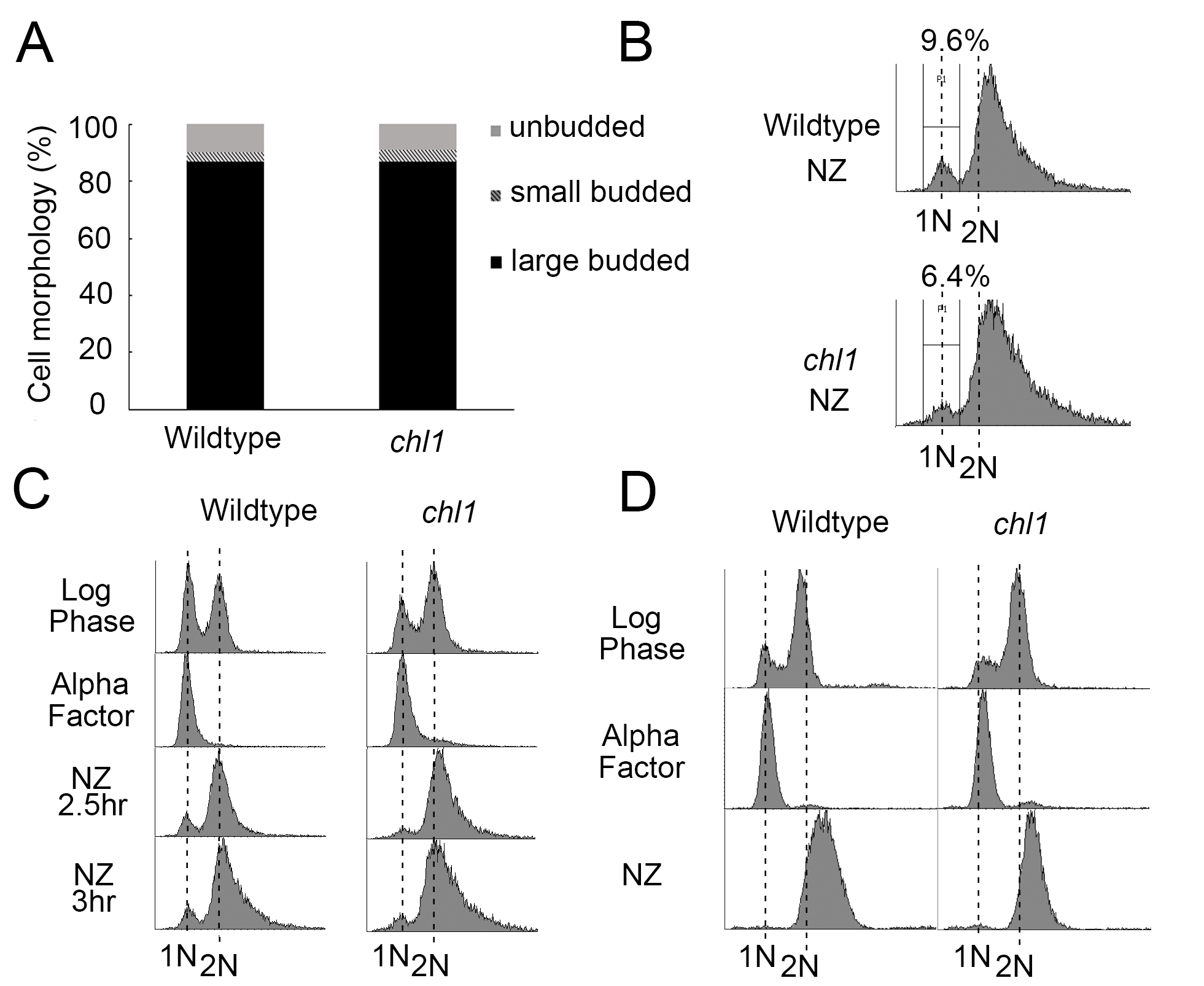

Supplement: S1 Fig — A) Morphology quantification for both nocodazole-arrested wildtype and chl1 mutant cells (N = 100 cells for each strain). B) 1N peak quantification for both wildtype and chl1 mutant cells arrested in nocodazole for 3 hours at 23°C. C) Flow cytometer data reveals DNA contents in wildtype and chl1 mutant cells after nocodazole arrest at 23°C for 2.5 hours and 3 hours. D) Flow cytometer data reveals DNA content in wildtype and chl1 mutant cells analyzed in Fig 1E and 1F. (TIF) [file pone.0227443.s001.tif]
